# Global patterns of migration of scholars with economic development

**DOI:** 10.1073/pnas.2217937120

**Published:** 2023-01-18

**Authors:** Ebru Sanliturk, Emilio Zagheni, Maciej J. Dańko, Tom Theile, Aliakbar Akbaritabar

**Affiliations:** ^a^Laboratory of Digital and Computational Demography, Max Planck Institute for Demographic Research, Rostock 18057, Germany

**Keywords:** high-skilled migration, bibliometric data, economic development, migration of scholars

## Abstract

We leverage metadata on over 36 million journal articles and reviews indexed by Scopus in order to estimate migration of scholars based on information on changes in their institutional affiliations over time. We produce a database of yearly international migration flows of scholars, for all countries from 1998 to 2017. We use the open-access database to provide descriptive evidence on the relationship between economic development and the emigration propensity of scholars. Statistical analysis using generalized additive mixed models reveals that emigration rates initially decrease as GDP per capita increases. Then, starting from around 25,000 dollars (2017 constant international dollars at purchasing power parity), the trend reverses and emigration propensity increases as countries get richer. This U-shaped pattern contrasts with what has been found in the literature for emigration rates for the general population and calls for theoretical frameworks to understand the heterogeneous responses of migration to development.

International migration and circulation of scholars is known to enhance research performance by facilitating knowledge recombination (1, 2) and is fundamental to scientific discovery, especially in its most innovative forms (3). Policymakers have also recognized the role of attracting “the best and the brightest” (4) in the global competition for talent, in order to favor economic growth and productivity. Indeed, scholars are a crucial group of innovators whose scientific breakthroughs have relevant economic effects in the countries of origin and destination. However, despite the relevance of scholarly migration and the need for deep comprehension of migration processes in order to devise sensible policy interventions, our ability to measure and monitor scholarly migration and mobility remains limited. One of the main factors that hinders the advancement of our understanding of migration of scientists, and the further development of theoretical frameworks, is a lack of high-quality and accessible data over an extended period of time.

Recently, the digitization of scholarly publications and their consolidation into large citation and abstract databases have opened up new opportunities for analyzing migration of scientists at scale (2, 5). In this study, we leverage bibliometric records from the Scopus database to estimate international relocations of scholars based on information on their changes in institutional affiliations. We produce the most comprehensive dataset on international migration of scholars, for the period from 1998 to 2017, and use it to provide descriptive evidence on the relationship between economic development and migration flows of scholars.

## Results

As a first step, we produced an innovative open-access database that contains counts of active academic scholars, migration flows, and migration rates for all countries that include Scopus-published scientists. This migration database, which is obtained by leveraging metadata of more than 36 million journal articles and reviews published over two decades, is a major resource to advance our understanding of migration of scholars. While the underlying data are proprietary, our approach generates anonymized aggregate-level datasets that can be shared for noncommercial purposes and that we are making publicly available for scientific purposes.

**Fig. 1.**
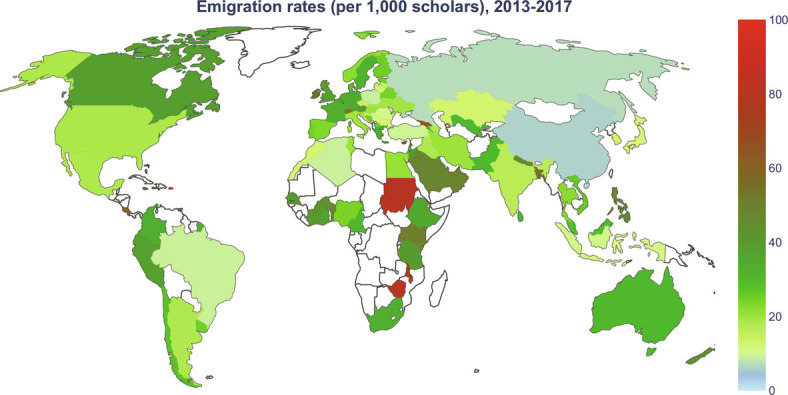
Average international scholarly emigration rates from 2013 to 2017, expressed as the number of emigrants per 1,000 active scholars per year. The map includes values for the 100 countries with the highest numbers of active scholars over time and with a general population size of at least 0.5 million people. Most countries have emigration rates below 40 per thousand scholars. Sudan, Malawi, and Zimbabwe, in Africa, and El Salvador and the Dominican Republic, in Latin America, have the highest rates. Other notable cases with high scholarly emigration rates include Canada in North America; Peru in Latin America; Switzerland and Ireland in Europe; Kenya and Uganda in Africa; Saudi Arabia, Oman, and Yemen in the Middle East; and Georgia, Bangladesh, and Nepal in Asia.

Fig. 1 shows average international scholarly emigration rates for the period 2013 to 2017. To avoid mapping values with potentially high stochasticity due to small sample sizes, here, we include estimates for the 100 countries with the highest numbers of active scholars over time and with a general population size of at least 0.5 million people. The countries where scholars have the highest emigration propensity are located in Africa. Globally, while most countries have emigration rates below 40 per 1,000 scholars, we also observed substantial heterogeneity. The map in Fig. 1 offers an illustrative example of the types of quantities for which we produced estimates and of the geographic coverage of the database.

After having observed substantial heterogeneity in scholarly migration rates across countries, we consider associations and descriptive trends. A central question in migration studies is how international migration responds to economic development. The answer to this question has huge implications for migration policy and for development assistance programs. From an economic point of view, income effects, as GDP per capita rises, would decrease the economic incentives to migrate. Migration theories, however, suggest an inverted U-shaped relationship, where economic development initially leads to higher international migration, which then decreases in later stages of economic development (6). This pattern of the so-called “migration transition” has been associated with a number of factors that include structural changes from an agricultural to an urban economy (7), demographic shifts, and higher capabilities and aspirations levels due to rising human capital (8, 9). Empirical analysis has also shown evidence that, as low-income countries economically grow, at the population level, emigration increases first and then declines (10, 11).

Migration transition theory leaves an open door for the international migration patterns of high-skilled professionals potentially diverging from the generic pattern (6). However, this is empirically underexplored due to lack of data. Here, we provide a descriptive assessment of the relationship between economic development and migration for published scholars, an important group of high-skilled migrants and innovators whose scientific breakthroughs have relevant economic effects on the countries of origin and destination (2). We observed that, for scholars, the relationship between migration and development is strikingly different from what has been observed for the general population (10, 11).

**Fig. 2.**
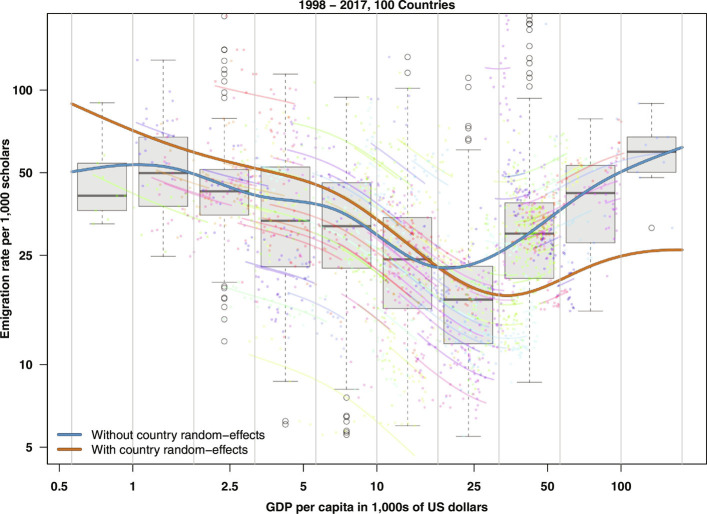
Scholarly emigration rates (1998 to 2017) as a function of GDP per capita [2017 constant international dollars at purchasing power parity (12)]. The combined data for each year and country are plotted as a box plot, while the country- and year-specific data are plotted using colored dots. Each color represents a separate country. The thick gray lines in the box plot represent median values, while the rectangles show the interquartile range. The blue thick line shows the years-averaged predicted emigration rates for a model that does not account for country-specific random effects (it does not include country-specific random intercept and random slope). The thick orange line shows predicted emigration rates excluding random effects and averaging along the year dimension for our main model that accounts for the country-specific random effects (*SI Appendix* for details). The thin colored lines show the predicted emigration rates for each country averaged along the year dimension and based on the same model as the one of the thick orange line.

Fig. 2 shows the descriptive relationship between emigration rates and GDP per capita (2017 constant international dollars at purchasing power parity) for the 100 countries included in Fig. 1. Observations based on a box plot indicate a U-shaped relationship. Further statistical analysis shows that both with and without controlling for the country random effects, emigration rates initially decrease as GDP per capita increases, and then, starting from around 25,000 dollars, the trend reverses and increases rapidly (see blue and orange lines in Fig. 2). Sensitivity analyses confirmed the robustness of the pattern. For instance, we tested changing the GDP measure and the number of countries included in the analysis. The results, the shape of the relationships, and the direction of the effect did not change (for details, *SI Appendix*). The U-shape pattern of the marginals shown in Fig. 2 is driven by the similar U-shape pattern of the effect of the smooth parameter of log GDP.

## Discussion

While it is well known that migration is a complex and heterogeneous process, the lack of high-quality data on migration flows, including for population subgroups, has hindered theoretical advances in our understanding of the heterogeneous response of different groups of migrants to both short-term shocks and long-term change. In this article, we developed a global database on international scholarly migration in order to assess emigration patterns and trends for this key group of innovators (1, 2).

Our empirical analysis focused on the relationship between emigration and economic development, indicating that the patterns in academic settings may be widely different from the population-level ones. We do not assess the underlying mechanisms, but we hypothesize that population heterogeneity matters for migration experiences. For example, the credentials of scholars might lead to lower barriers to entering rich countries. Moreover, scholars are more likely to have enough financial resources, or enough access to credit, for an international move, than the general population in a low-income country. They are also more likely to have an international network of colleagues and have developed migration aspirations. In other words, scholars in low-income countries are, on average, more capable to move than other segments of the population (8, 9). In addition, they have incentives to move, including large differentials with richer countries in terms of scientific infrastructure and standards of living. As countries grow economically, the incentive for scholars to migrate may initially decrease, as local academic opportunities grow, in part as a result of the expansion of the higher education system to face the demands of a changing economy and labor market. With economic development, the reduced propensity to migrate may also derive from circumstances such that emigration flows increase at a slower pace than the increase in active academic scholars, which form the denominators for rate calculations.

The academic environment in the richest countries is such that universities and research institutions that compete for top places in world rankings increasingly spur international mobility. Attracting more international members (both students and faculty) is considered a performance indicator for institutions of higher education (13). Emphasis on international collaboration and brain circulation at academic institutions thus provide fertile ground for increased scholarly migration. Spending part of the academic career abroad is regarded as essential for top researchers (1, 2), and scientists in high-income countries also have more resources in order to be able to be internationally mobile. Thus, higher emigration rates of scholars from rich countries should be considered in view of larger forces that lead to the circulation of talent, including also return migration, with positive effects on the whole science system.

This study creates a bridge between the fields of demography and migration studies on the one hand and science of science on the other. However, it does so not without limitations. From a data perspective, the emigration counts do not necessarily refer to nationals that leave a given country (5). Indeed, scholars who emigrate in this setting may also be return migrants (14), who go back to their countries of nationality after completing their studies and/or their long-term stay abroad. Further refinements of estimation procedures, possibly by linking additional sources, or in combination with ad hoc surveys, could resolve definition-based ambiguities in future research.

This study does not use a causal framework. The relationships observed are relevant and robust. Nonetheless, the observed regularities remain statistical associations that cannot be interpreted in a causal way. It is beyond the scope of this article to achieve causal explanations. In the study, we focus on macrodemographic trends. However, the underlying microdata that we use is structured essentially as a repeated panel where scientists are followed throughout their careers, as long as they remain active in terms of publishing. Future studies can leverage these rich microlevel data to move toward more causal explanations of underlying mechanisms that explain observed patterns.

## Materials and Methods

In this study, we leveraged bibliometric data to detect migration events of published scholars, to aggregate them, and to assess the relationship between scholarly migration and economic development. We used a 2020 snapshot of Scopus data maintained by Elsevier and provided to us by the German Competence Centre for Bibliometrics via the Max Planck Digital Library. We assessed changes in residence on the basis of changes in academic affiliation on an annual basis. This approach is in accordance with the definition of long-term emigrant as a person who changes his/her country of residence for a period of at least 1 y (15). For each year, we assessed the residence of the scholar as the country of primary affiliation with the largest number of publications for that year. Economic indicators come from the World Bank’s GDP per capita data, PPP (constant 2017 international dollars). In the statistical analysis, we used the generalized additive mixed model (GAMM) approach.

Our GAMM includes the following terms: smooth function of year *s*(*year*) and smooth function of log GDP *s*(log GDP) as fixed terms, and two terms related to the country effect: random intercept *s*(*country*) and random slope *s*(*country*, log GDP). The model formula reads:

$$\begin{array}{cc} log(E(Y)) & =\beta_{0}+s\left(\log\;\mathrm{GDP}\right)+s\left(year\right)+s\left(country\right)\\ {}[1pt] & \;+s(country,\log\;\mathrm{GDP})+\log\;\mathrm{exposures}, \end{array}$$

where *Y* is the emigration count and *β*_0_ is the intercept. More detailed information on detecting migration events, the statistical analysis, and supporting sensitivity analyses, can be found in *SI Appendix*.

## Supplementary Material

Appendix 01 (PDF)

## Data Availability

Some study data available. In our research we used the bibliometric data through the German Competence Centre for Bibliometrics (Kompetenzzentrum Bibliometrie, grant number 16WIK2101A, https://bibliometrie.info/) and the access they granted to the Max Planck Digital Library. Sharing the individual-level original raw data with third parties is against the data usage agreement. We curated an anonymized, aggregate version of the data, that removes the commercial value of the data, but maintains its scientific value. Scripts and data to replicate our results are publicly accessible on GitHub under https://github.com/MaciejDanko/Global-Migration-of-Scholars-code-and-data-repositry-for-PNAS.
