# Supplementary material for: Global patterns of migration of scholars with economic development
Source: Proc Natl Acad Sci U S A. 2023 Jan 18;120(4):e2217937120. doi: 10.1073/pnas.2217937120 (PMC9942787; doi:10.1073/pnas.2217937120)
Supplement: Supplementary file 1 — Appendix 01 (PDF) [file pnas.2217937120.sapp.pdf]

Supporting Information for

Global patterns of migration of scholars with economic development

Ebru Sanliturk, Emilio Zagheni, Maciej J. Dańko, Tom Theile, and Aliakbar Akbaritabar

Corresponding Author: Emilio Zagheni
E-mail: zagheni@demogr.mpg.de

This PDF file includes:

Supporting text
SI References

Supporting Information Text

1. Extended Methods

A. Pre-processing bibliometric data. To produce a global database of scholarly migration, we used bibliometric data. Bibliometric data include information that is extracted from scholarly publications in scientific journals (1–4). This varies from substantive data (i.e., title, abstract, manuscript text and keyword of the publications) to meta-data accompanying these publications (i.e., authors' names, affiliation addresses, references list, and publication year).

The Scopus database, maintained by Elsevier, contains metadata about more than 77 million publications, on a global scale (5). For our analyses, we limit the publications to the subset of 36.2 million *articles* and *reviews* from 1996. We made this choice because articles and reviews have been found to have the highest level of accuracy for metadata (6).

The selected subset of data we use in this research includes the names and affiliations of 16 million author profiles disambiguated by Elsevier (7). Authors are identified across different publications with an author_id. This author_id identifies all publications of a single author in 94.4% of cases (recall) and has a precision of 98.1%. This means that records of two different authors could be merged by mistake under one author_id only in 1.9% of the cases (7).

The affiliation includes the address and the country, and in some cases an affiliation_id, which identifies unique institutions, even if their names are spelled differently. The full address of institutions is available only in 87% of the records, but the country where the institution is located is available in 99% of the records (7).

Using bibliometric databases and re-purposing them to identify scholarly mobility events offers unprecedented opportunities. It also comes with limitations that we would like to acknowledge. In addition to potential issues with author name disambiguation discussed above, some assumptions are needed when considering the relationship between publication dates and the time when mobility events occur. As the publication process often takes time (8), we may be measuring mobility with a certain lag. It is important to be aware of this: even though it would not affect trajectories of trends, it may shift them slightly. Also, considering country of affiliation as the country of residence of scholars entails an underlying assumption that scholars' working and living locations are the same. It is likely that, within countries, some scholars live relatively far from where their institution is located. At the country-level, given existing legal constraints related to working in one country and establishing residence in a different one, we expect the faction of scholars in this situation to be very small and not consequential for our results. The country of affiliation for the first publications is considered the country of academic origin: this could be the country where the scholar studied or where they had their first academic position. In other words, country of origin should be intended as the country where the scholar received the last major component of their academic training. Finally, the data are limited to those scholars who have actively published in scientific journals indexed by Scopus. This means that the population of scholars considered may be biased towards those who publish in English-speaking journals (1–3). This also means that we may be considering the most dynamic group of scholars who are active and publish in internationally visible journals.

B. Estimating emigration and count of scholars. Migration counts are obtained by aggregating all migration events estimated at the micro-level, based on changes in the country of institutional affiliation of authors. After pre-processing the raw authorship records, we determined the country of residence for every author and every year. More specifically, we considered all the affiliation countries of an author_id in one year. If there was more than one country, we chose the mode of all countries. If there was more than one mode, we checked whether one of the modes was the previous country of residence and selected that country as the country of primary residence, if that were the case. If it were not, we chose one of the mode countries randomly.

To determine migration events, our algorithm went through the years and logged a migration event when the country of residence changed. We assume a up to *two years* preparation time for all publications to cover disciplinary differences in publication delay (8). If there were gaps in publication years (e.g., for authors who do not publish every year), we assumed that the country of residence of the author (for up to two years before the year of publication) was the same as the country of residence for the year of publication. Then we considered the year when a modal affiliation changed as the migration year.

Once we had detected all migration events, we aggregated them by country and by year into emigration counts. In order to generate measures of exposure (i.e., the denominators for migration rates, or the population size of researchers per country and year), and to deal with missing observations, we assumed that an author who did not publish in a particular year was still part of the population of active scholars if they published one or two years before. In addition, we excluded from the denominators/exposure the authors who had only *only* one indexed publication during their entire career.

C. Statistical analysis. The Generalized Additive Mixed Models (GAMM (9)) used in our study are an extension of the Generalized Linear Mixed Models (GLMM) that allow one to model non-linear relationships using penalized smooth functions of selected predictor variables (smooths). Emigration rate was modeled using count data models with offset set to the log number of scholars in a specific country and for a given year (exposures approximation). We used the negative binomial distribution (with log link function) because it can account for overdispersion, which is a common problem that arises when fitting standard Poisson models. The model was fitted in R (10) using the *gam*() function of the *mgcv* package (9).

Our main model used in the manuscript includes the smooth function of year ($s(year)$) and smooth function of log GDP ($s(log\ GDP)$) as fixed terms. The random intercept ($s(country)$) and random slope ($s(country,\ log\ GDP)$) represent the country effect. Fixed smooth terms used P-spline ("ps") as the smooth basis, and random effects were modeled using random smooths ("re" smooth basis, (9)).

Simpler models were also tested by dropping some of the terms. However the model with all terms was the most parsimonious one according to the AIC (Akaike Information Criterion (11)). We applied a log transformation of GDP because we assume that a change in GDP with lower values of this variable has a much greater impact on migration rates than the same change in GDP with higher values. We fitted the model to the 100 countries with the highest average number of scholars between 1998 and 2017, and with an average population size greater than half a million.

To predict the emigration rates we used the R function *predict.gam*(). We used year-averaged predicted log emigration rates with excluded random effects ("exclude" parameter in *predict.gam*() function). The model predictions can be described by the function:

$$\overline{R} = \beta_0 + s(log\, GDP) + \frac{1}{N} \sum_{year=1998}^{2017} s(year),$$

where $\overline{R}$ is the year-averaged log emigration rate, s are the fitted smooths, and N = 2017-1998+1 is the number of years.

2. Sensitivity analyses

The basic model uses data from the 100 countries with the largest numbers of scholars. We also checked whether fitting models on a smaller number of countries (90, 80, 70, 60, 50, 40, 30, and 20) has an impact on the consistency of the results. This sensitivity analysis shows that the resulting GDP-mortality U-pattern and the year-specific patterns are qualitatively consistent when at least 40 or more countries are included in the analysis.

Furthermore, we tested our model for different measures of GDP, to check the robustness of our results. In the main model we used the World Bank's *GDP per capita, PPP* (*constant 2017 international dollars*) (12), which accounts for the relative cost of living, inflation rates (Purchasing Power Parity, PPP) and price indexes, as it is measured in constant international dollars (2017 as the reference year). For the sensitivity analysis we considered also two other options: (1) GDP measured in constant 2010 dollars without accounting for PPP and (2) current GDP that accounts for PPP. In the latter case, the U pattern (orange line) is attenuated, but emigration rates at the highest GDP values do not decrease further: they remain fairly stable.

3. Replication materials

The data and code used for the analysis and plotting of the figures are available via GitHub repository:
https://github.com/MaciejDanko/Global-Migration-of-Scholars-code-and-data-repositry-for-PNAS
and its clone repository at OSF: https://osf.io/238zn/

References

1. G Laudel, Studying the brain drain: Can bibliometric methods help? *Scientometrics* **57**, 215–237 (2003).
2. HF Moed, G Halevi, A bibliometric approach to tracking international scientific migration. *Scientometrics* **101**, 1987–2001 (2014).
3. HF Moed, M Aisati, A Plume, Studying scientific migration in scopus. *Scientometrics* **94**, 929–942 (2013).
4. R Kashyap, et al., Digital and Computational Demography, (SocArXiv), Technical report (2022).
5. Scopus, Content coverage guide (2020).
6. Kompetenzzentrum bibliometrie (competence centre for bibliometrics) (https://bibliometrie.info/) (2021) Accessed: 2021-09-24.
7. J Baas, M Schotten, A Plume, G Côté, R Karimi, Scopus as a curated, high-quality bibliometric data source for academic research in quantitative science studies. *Quant. Sci. Stud.* **1**, 377–386 (2020).
8. BC Björk, D Solomon, The publishing delay in scholarly peer-reviewed journals. *J. Informetrics* **7**, 914–923 (2013).
9. SN Wood, *Generalized Additive Models: An Introduction with R.* (Chapman and Hall/CRC), 2 edition, (2017).
10. R Core Team, *R: A Language and Environment for Statistical Computing* (R Foundation for Statistical Computing, Vienna, Austria), (2020).
11. KP Burnham, DR Anderson, Multimodel inference: understanding AIC and BIC in model selection. *Sociol. Methods & Res.* **33**, 261–304 (2004).
12. WB World Development Indicators, Gdp per capita, ppp (constant 2017 international dollars) (2021) Accessed: 2021-09-21.
